# Highly Discriminative Physiological Parameters for Thermal Pattern Classification

**DOI:** 10.3390/s21227751

**Published:** 2021-11-21

**Authors:** Laura Benita Alvarado-Cruz, Carina Toxqui-Quitl, Raúl Castro-Ortega, Alfonso Padilla-Vivanco, José Humberto Arroyo-Núñez

**Affiliations:** Computer Vision Laboratory, Universidad Politécnica de Tulancingo, Tulancingo de Bravo 43629, Mexico; laura.alvarado.1631026@upt.edu.mx (L.B.A.-C.); raul.castro@upt.edu.mx (R.C.-O.); alfonso.padilla@upt.edu.mx (A.P.-V.); humberto.arroyo@upt.edu.mx (J.H.A.-N.)

**Keywords:** breast thermography, heat source parameters, feature extraction, infrared imaging, D-I-R model

## Abstract

Infrared Thermography (IRT) is a non-contact, non-intrusive, and non-ionizing radiation tool used for detecting breast lesions. This paper analyzes the surface temperature distribution (STD) on an optimal Region of Interest (RoI) for extraction of suitable internal heat source parameters. The physiological parameters are estimated through the inverse solution of the bio-heat equation and the STD of suspicious areas related to the hottest spots of the RoI. To reach these values, the STD is analyzed by means: the Depth-Intensity-Radius (D-I-R) measurement model and the fitting method of Lorentz curve. A highly discriminative pattern vector composed of the extracted physiological parameters is proposed to classify normal and abnormal breast thermograms. A well-defined RoI is delimited at a radial distance, determined by the Support Vector Machines (SVM). Nevertheless, this distance is less than or equal to 1.8 cm due to the maximum temperature location close to the boundary image. The methodology is applied to 87 breast thermograms that belong to the Database for Mastology Research with Infrared Image (DMR-IR). This methodology does not apply any image enhancements or normalization of input data. At an optimal position, the three-dimensional scattergrams show a correct separation between normal and abnormal thermograms. In other cases, the feature vectors are highly correlated. According to our experimental results, the proposed pattern vector extracted at optimal position a=1.6 cm reaches the highest sensitivity, specificity, and accuracy. Even more, the proposed technique utilizes a reduced number of physiological parameters to obtain a Correct Rate Classification (CRC) of 100%. The precision assessment confirms the performance superiority of the proposed method compared with other techniques for the breast thermogram classification of the DMR-IR.

## 1. Introduction

**Figure 1:**
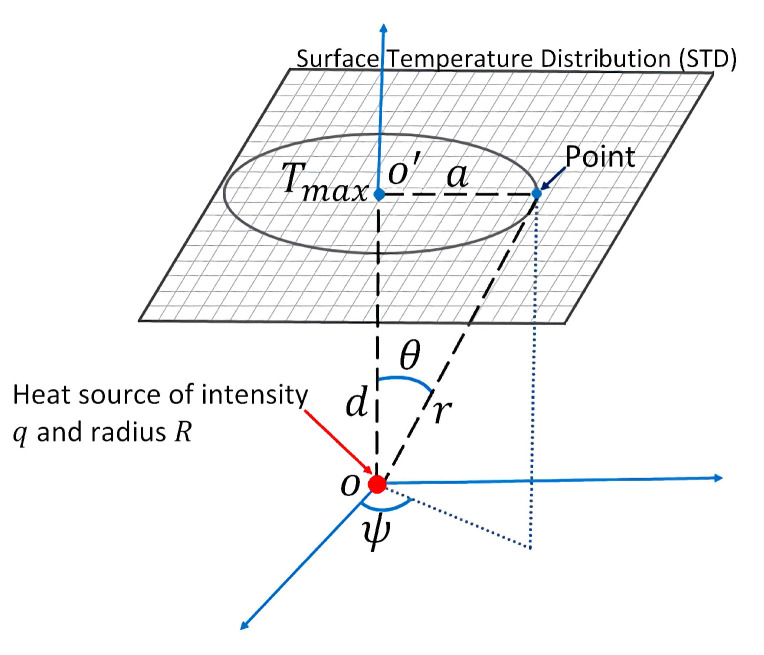
Scheme of the theoretical model of an internal heat source with depth *d*, intensity *q*, and radius *R*.

It is well known that body temperature is a standard indicator of health status in humans. IRT records radiant energy that is emitted by the human body at wavelengths between 2 μm and 14 μm. Infrared energy is a function of skin temperature with an average emissivity of 0.97–0.99 [1], then an IR image gives the temperature distribution of the human body. In 1963, Lawson and Chughtai [2] reported the use of surface temperature measurements as a possible tool for breast cancer diagnosis. They found that the diseases make the thermal gradient vary in this area, and subtle thermal abnormalities can be related to a particular disorder. Thenceforth, research on medical applications of infrared technology has been published, and different databases have been created, such as the public DMR-IR [3,4]. The first one on the quantitative relationship describing the temperature distribution in human tissue and considering blood flow effects based on the continuum theory was presented by Harry H. Pennes [5]. Even more, a hot spot in the surface temperature distribution, related to tumor tissue, has been modeled as a point heat source [6]. A model of the internal heat source is illustrated in Figure 1. Several papers [6,7,8,9,10,11,12] point out that an inverse solution of the bio-heat equation can be used for the estimation of a point heat source parameters: intensity *q*, depth *d*, position *a*, and radius *R*, from the STD of the thermal input data. Table 1 describes the thermal and biological parameters used in this paper.

Guilian Shi et al. [8,9] make use of the biological heat transfer model and the fitting method of Lorentz curve for the extraction of the above-mentioned physiological parameters. The distribution and tendency of the q−r curve are related to the typification of breast lesions. They established a criterion based on the angle of inclination θ of the q−r curves to solve this problem. Nevertheless, the q−r curve shows ripples or oscillations that make it difficult to establish a single angle value. Hossain et al. [6] developed a methodology for the heat source parameters calculation, and it is based on the D-I-R measurement model. This model has been implemented numerically to calculate the parameters. Rastgar-Jazi et al. [10] establish a range for intensity *q* depending on the *a* position when the D-I-R model is applied. In this context, mostly numerical and simulated analyses have been done [6,7,10,11,12]. In general, most of the authors establish a range of values for *q* [10,11,12]. It leads to ambiguous risk decisions when a single value for *q* is defined. Even more, a unique value of *q* in a well-defined *a* position is needed to classify normal and abnormal breast thermograms. Some works [8,9] make use of the angle of the mentioned above q−r curve for the diagnosis of breast diseases. Then again, the trend of the curve also causes uncertainty when a unique angle must be defined. Additionally, the breast thermogram categorization can use other thermal characteristics as the temperature increases for the tissue [13], but it is not a determining feature since the maximum temperature in the thermal breast data can be greater than 3 °C and be labeled as healthy.

**Figure 2:**
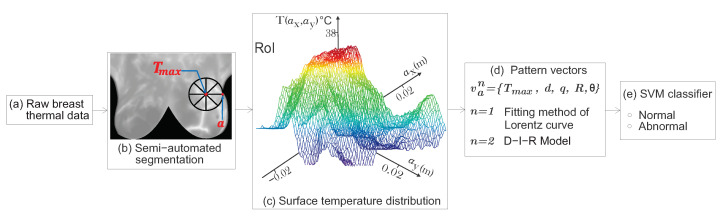
Flowchart of the proposed method. (**a**) Thermal data were obtained from the DMR-IR. (**b**) A well-defined RoI is delimited at an optimal radial distance *a*. As we can see, the RoI encircle the temperature area to be analyzed. (**c**) Surface temperature distribution related to the hottest spot of the RoI. (**d**) A proposed highly discriminative pattern vector is composed by the physiological parameters from the point heat source. (**e**) Classification step using SVM.

Therefore, based on the solution of the inverse heat conduction problem, we propose a highly discriminative pattern vector composed of physiological parameters to classify normal and abnormal thermograms. In addition, a well-defined RoI from the input STD is analyzed to extract the internal heat source parameters. Through SVM, the optimal radial distance *a* of the RoI is determined [14]. The proposed methodology was applied to 87 breast thermograms that belong to the DMR-IR [3,4]. The proposed pattern vector is composed of the physiological parameters $v_{a}^{n}=${$T_{max},d,q,R,\theta$} and is used for the classification by means of SVM. The three-dimensional feature space proofs the discrimination power of the proposed pattern vector, allowing the correct separation of both classes. At an optimal radial distance *a* of the RoI and through (1) the fitting method of Lorentz curve and (2) the D-I-R model, a CRC of 90.80% and 100%, respectively, are obtained using the physiological descriptors. The applied methodology allows reducing the rate of false-positives or false-negatives. Figure 2 shows the research methodology flowchart.

In this manuscript, the sections are organized as follows: The raw thermal data and the semi-automated segmentation algorithm are reported in Section 2. Moreover, a review of the methods for estimating physiological parameters based on an inverse solution of the bio-heat equation from the input surface temperature matrix is given. The classification process of a set of ϵ=87 breast thermograms belonging to the DMR-IR in normal and abnormal classes using SVM is also described. In Section 3, the experimental results obtained by extracting the physiological parameters of a well-defined RoI from the breast thermograms are presented. Furthermore, the three-dimensional feature space of the above-mentioned parameters and the classification percentages are given. Section 4 is dedicated to discussing the results. Finally, the conclusions are summarized in Section 5.

## 2. Materials and Methods

### 2.1. Image Database

The public DMR-IR has been used to evaluate the performance of statistical methods to classify breast thermograms into normal and abnormal [3,4,15,16]. This database contains thermal images with their corresponding clinical data. It consists of 287 volunteers, of which 244 are reported as healthy, 39 sick, and 4 with an unknown diagnosis [4]. The diagnosis in people was made by mammography and/or biopsy. Frontal images are considered for this analysis. Table 2 shows the FLIR thermal camera specifications used by Silva et al. [4] to capture breast thermograms. The FLIR camera has a 24${}^{\circ }$ standard lens with focal length f=75 mm [17]. The standard distance between the thermal camera and the patient is 1 m [4]. The object height is given by $l_{o}=\frac{2.16\;\mathrm{cm}}{7.5\;\mathrm{cm}}\left(100\;\mathrm{cm}\right)=28.8$ cm [18]. Therefore, the pixel size in the object plane is $S_{p}=\frac{l_{o}}{N}=0.06$ cm.

### 2.2. Segmentation of Breast Thermograms

**Figure 3:**
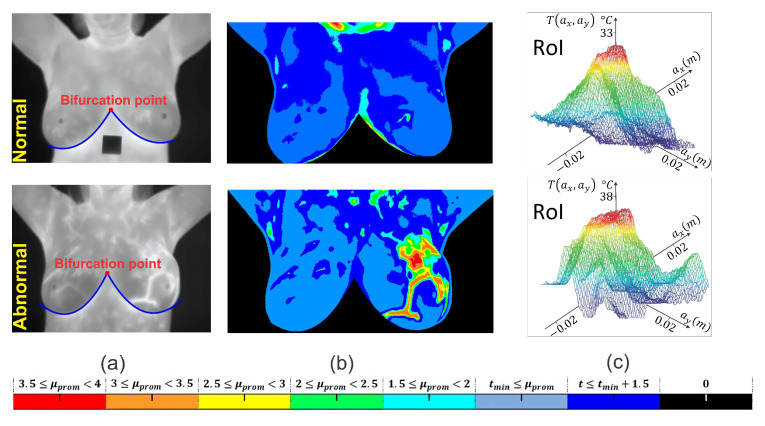
RoI delimitation process. (**a**) Segmentation procedure based on the detection of the inframammary line by a polynomial curve fitting. (**b**) Visualization of a clustered thermal pattern through thermal gradients. (**c**) Centered RoI around the hottest point with radial distance *a*.

A semi-automated segmentation algorithm is implemented employing a cubic degree polynomial curve fitting [19,20]. A binary image mask of size M×N pixels is created to eliminate the area under the inframammary curves detected by the polynomial curve fitting [21]. Neither image enhancements nor normalization of input data is done. Once the breast image is segmented, the temperature changes are clustered using the thermal gradient, thus facilitating the location of the RoI, which is shown in Figure 3b. The hottest region of thermal images is defined as the RoI with a radial distance *a*, and it is shown in Figure 3c.

### 2.3. Heat Source Model: A Mathematical Review

The Pennes bio-heat equation is used to analyze the interior temperature distribution of biological tissue. It is given as [5],

(1) $$\rho c\frac{\partial T}{\partial t}=▽\left(k\cdot ▽T\right)+w_{b}\rho_{b}c_{b}\left(T_{a}-T\right)+Q_{m}.$$

Table 1 describes each variable in Equation (1). The terms $w_{b}\rho_{b}c_{b}\left(T_{a}-T\right)+Q_{m}$ are merged to be the internal heat source. A solution of Equation (1) is given by [6,7,12],

(2) $$T=T_{e}+\frac{q}{4\pi h_{0}r^{2}}.$$

The maximum temperature $T_{max}$ is obtained when r=d, which is the temperature at the point $O^{\prime }$ in Figure 1. Suppose *a* is the distance from point $O^{\prime }$ to an arbitrary point on body surface, then $r^{2}=d^{2}+a^{2}$. Therefore,

(3) $$T\left(a\right)=T_{e}+\frac{q}{4\pi h_{0}\left(d^{2}+a^{2}\right)}.$$

**Figure 4:**
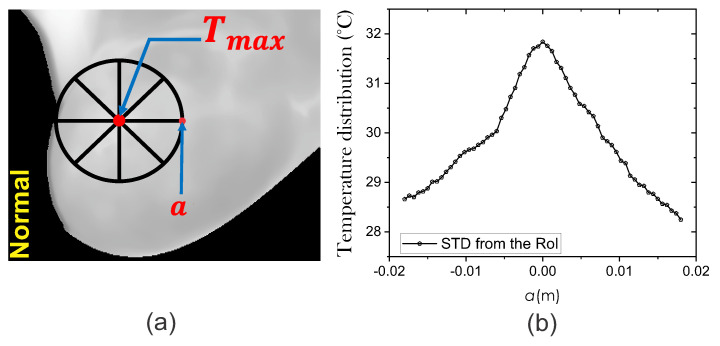
(**a**) The extraction process of the STD using thermal input data. (**b**) Mean temperature distribution around the hottest point with radial distance *a*.

Abnormal tissue can be modeled as a spherical heat source with intensity *q*, radius *R*, and depth *d* [6,10]. Then,

(4) $$T\left(a\right)=T_{e}+\frac{q}{4\pi h_{0}\left[{\left(d+R\right)}^{2}+a^{2}\right]},$$

where T(a) is the temperature at any arbitrary point *a* on the STD of the thermal input data. The temperature distribution T(a) is obtained from the thermal input data at each side of the maximum temperature point $T_{max}$. Thermal vectors are acquired in all four directions and are represented as straight lines of radial distance *a* as shown in Figure 4a. In this work, the four thermal vectors are averaged. Figure 4b shows the mean surface temperature distribution.

#### 2.3.1. Fitting Method of Lorentz Curve

**Figure 5:**
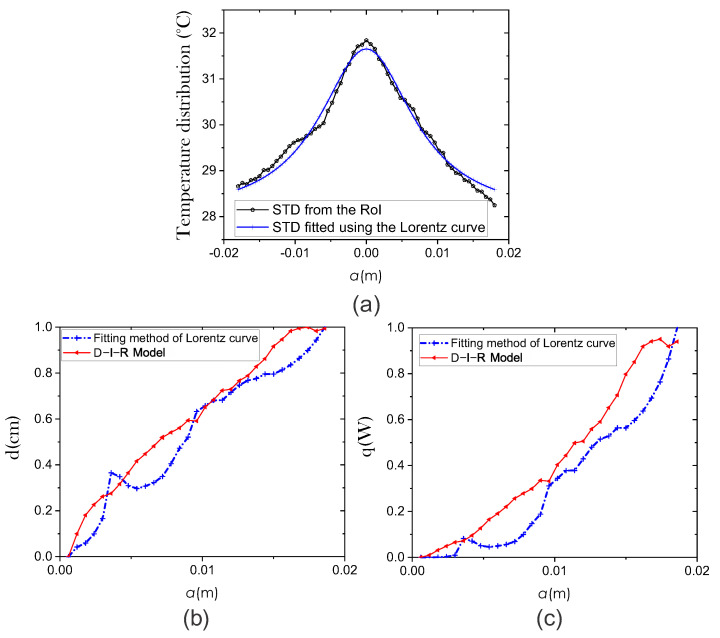
Comparison of the efficiency of two methods for extracting physiological parameters: fitting method of Lorentz curve (blue line) and the D-I-R model (red line). (**a**) STD fitted using the Lorentz curve method. We use the coefficient of determination (R-squared) to quantify the fitting between the surface temperature curve and the Lorentz curve. Estimation of physiological parameters (**b**) Depth *d* and (**c**) Intensity *q*.

As is shown in Figure 5a, the experimental surface temperature distribution fits the Lorentz curve $y=A/\left(a^{2}+w^{2}\right)+y_{0}$ to obtain information of an internal heat source [8]. We have implemented a plugin in Matlab to obtain the STD fitted by the Lorentz curve method [22]. In this way, the estimated depth and intensity are acquired as d=w and $q=4\pi h_{0}A$, for advisability, set $4\pi h_{0}=1$. As is shown in Figure 5c, the angle of the q−a curve can be obtained by θ(a)=arctan[q(a)/a].

#### 2.3.2. D-I-R Model

The heat source parameters are obtained through the D-I-R model as [6],

(5) $$d\left(a\right)=\frac{a\sqrt{\left(T\left(a\right)-T_{e}\right)}}{\sqrt{T_{max}-T\left(a\right)}},$$

(6) $$q\left(a\right)=4\pi h_{0}\frac{\left(T\left(a\right)-T_{e}\right)\left(T_{max}-T_{e}\right)}{T_{max}-T\left(a\right)}a^{2},$$

and

(7) $$R=\sqrt[{3}]{\frac{q}{Q_{m}A_{t}}},$$

for $Q_{m}=418.6$ W/m^3^, $h_{0}=8.77$ W/m^2^ · °C, and volume of cell is $A_{t}=1$ μm [6]. The d−a and q−a curves are obtained using Equations (5) and (6) and shown in Figure 5b,c, respectively.

Then, a set of physiological parameters is estimated at different positions a=−0.018:0.0006:0.018 m and through the fitting method of Lorentz curve (n=1) and the D-I-R model (n=2). In this way, two pattern vectors are defined as $v_{a}^{n}=${$T_{max},d,q,R,\theta$} for n=1,2 by using the extracted physiological parameters. The classification step make use of the physiological pattern vectors.

### 2.4. Thermal Pattern Classification Using SVM

**Figure 6:**
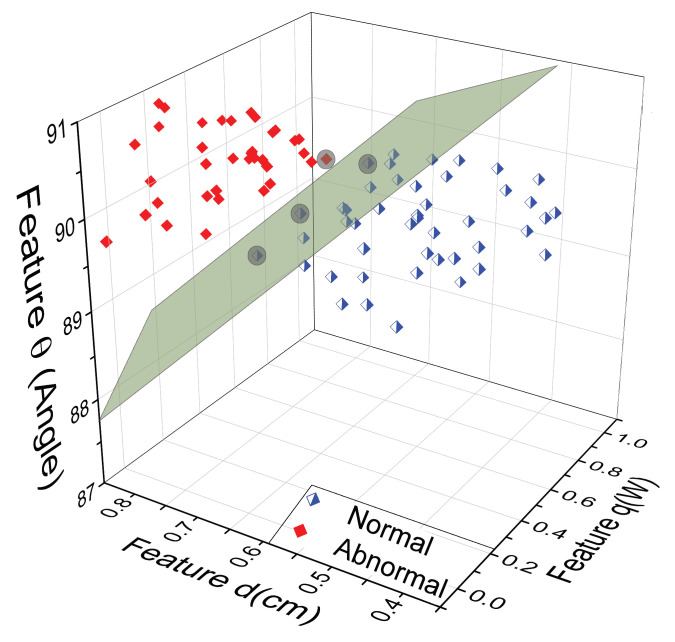
Three-dimensional space using the proposed physiological parameters $v_{a}^{n}\left(i\right)=\;${θ,d,q} obtained through the D-I-R model, corresponding to n=2 at a given position *a*. The support vectors define the margin’s greatest separation between the normal and abnormal classes.

Cortes and Vapnik in 1995 developed the Support Vector Machines [23]. SVM has multiple applications and can be used to solve classification problems. This section describes the classification process of a set of ϵ=87 breast thermograms from the DMR-IR into normal and abnormal classes. Generally, this stage involves two datasets: training and testing. We use a *K*-fold cross-validation with K=10. Each one is a composite of data instances. The training set {$x_{i}\left(a\right)$, $y_{i}$} for i=1…ϵ contains the several features or “attributes” accompanied of target class values or “labels”. The proposed pattern vectors or attributes $x_{i}\left(a\right)=v_{a}^{n}\left(i\right)=${$T_{max},d,q,R,\theta$} for n=1,2 are composed of the physiological parameters at a given position *a* with |x(a)|=χ=5. There are 49 thermograms labeled as healthy and 38 labeled as unhealthy. The principal objective of SVM is to find a computationally efficient way to produce a model to predict target class values given a testing dataset with attributes only. As is shown in Figure 6, a separating hyperplane into a χ-dimensional feature space must be implemented.

For the classification task, we use the model formulation *C*-classification given as [24],

(8) $$\begin{array}{cc} \min_{\alpha } & \frac{1}{2}\alpha^{T}B\alpha -e^{T}\alpha \\ s.t. & 0\leq \alpha_{i}\leq C,\;i=1,\ldots ,l,\\ & y^{T}\alpha =0. \end{array}$$

To reduce some error measure above the training data, $\alpha_{i}$ are the weights from the *i*-th hidden unit to the output unit, *b* is the bias, **e** is the unity vector, *C* is the upper bound, $B_{i,j}\equiv y_{i},y_{j}P\left(x_{i}\left(a\right),x_{j}\left(a\right)\right)$, $P\left(x_{i}\left(a\right),x_{j}\left(a\right)\right)\equiv \varphi {\left(x_{i}\left(a\right)\right)}^{T}\varphi \left(x_{j}\left(a\right)\right)$, and φ is the mapping function [25].

Assuming that the nonlinear separation limit can be linearized in a larger-dimensional feature space using a mapping method: $\varphi :R^{\chi }↣H:R^{\chi +s}\Rightarrow x↣\varphi$ (*x*), where *v* is the increased dimension of *H* space [25]. The nonlinear SVM classifier is given as [25,26],

(9) $$f\left(x\right)=\mathrm{sgn}\left[\sum_{i=1}^{\epsilon }\alpha_{i}y_{i}P\left(x_{i}\left(a\right),x_{j}\left(a\right)\right)+b\right],\;\alpha_{i}>0.$$

The Radial Basis Function (RBF) is the kernel, defined as $P\left(x_{i}\left(a\right),x_{j}\left(a\right)\right)=exp(-\gamma ∥$$x_{i}\left(a\right)-x_{j}\left(a\right){∥}^{2})$, where γ is gamma function, $x_{i}\left(a\right)$ are the training vectors, and $x_{j}\left(a\right)$ are called support vectors.

## 3. Results

**Figure 7:**
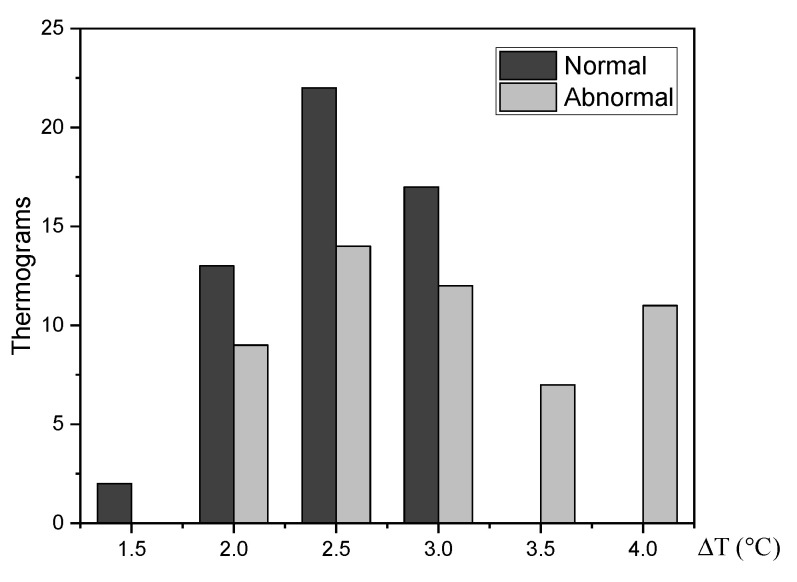
The temperature at the vicinity of affected tissue is about 2 °C higher than normal tissue.

The STD for a set of 87 thermograms, 49 normal and 38 abnormal of the DMR-IR, is analyzed [3]. Abnormal thermal patterns that may be linked to breast lesions are highlighted by an increase in the temperature of the affected tissue [13]. An increase $\Delta T=T_{max}-T_{mean}$ in surface temperature is calculated for each thermogram. The Figure 7 shows that all the abnormal thermograms presenting temperature increases ΔT≥2 °C. Hence, the maximum temperature $T_{max}$ point of the RoI will be part of the pattern vector for the thermogram categorization.

### Extraction of the Input Heat Source Parameters

**Figure 8:**
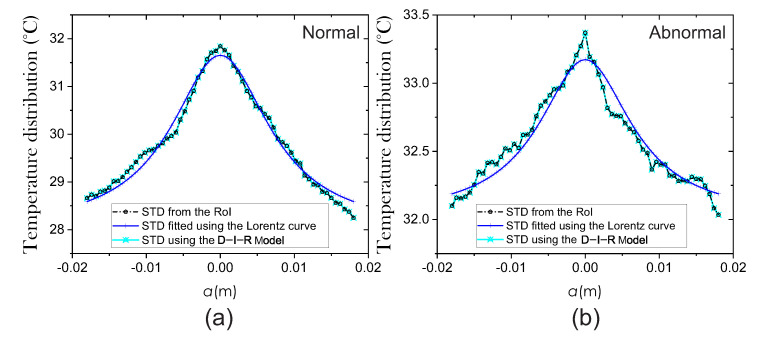
Surface temperature distribution from (**a**) normal and (**b**) abnormal breast thermograms.

The surface temperature distribution T(x), environment temperature $T_{e}=22$ °C [4], and maximum temperature $T_{max}$ at the RoI are needed to extract the internal heat source parameters. For extraction of parameters, we applied the methodology mentioned in Section 2. Firstly, to measure the correlation between the STD from the RoI and the STD fitted by the Lorentz curve, we use the coefficient of determination *R*-squared [27]. In the same way, the correlation between the STD estimated using the Equation (4) of the D-I-R model and the STD of the RoI is measured. Therefore, the mean *R*-squared values corresponding to the 87 thermograms are 0.87 and 0.99, respectively. Figure 8 shows the estimated STD employing the two methods.

**Figure 9:**
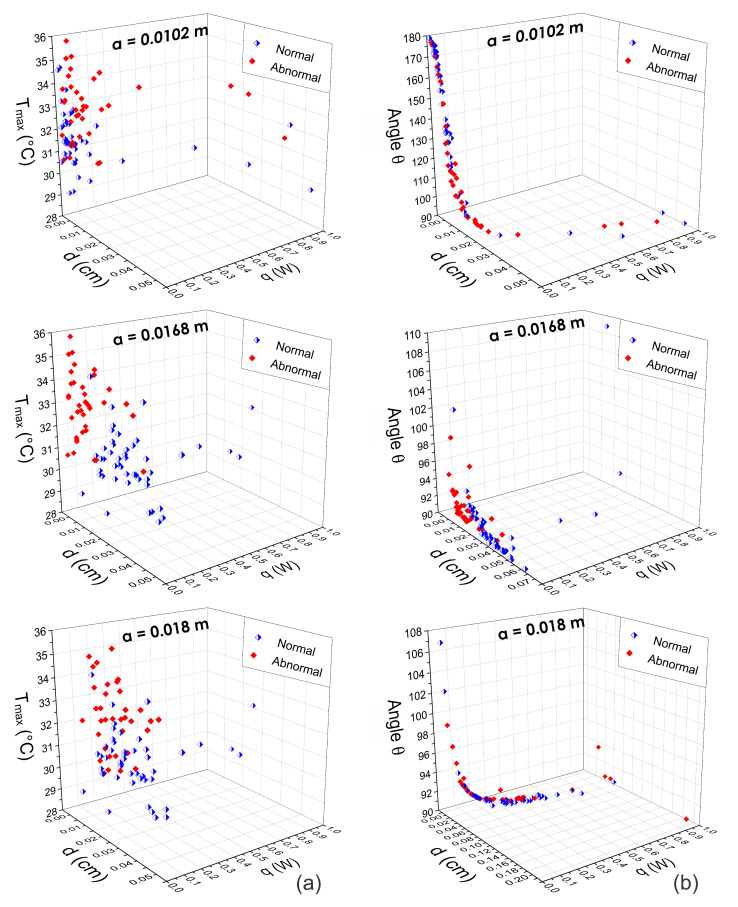
Three-dimensional scattergrams using the physiological parameters obtained by means of the fitting method of Lorentz curve at different positions a=0.0102 m, a=0.0168 m, and a=0.018 m. Column (**a**) corresponds to the pattern vector $v_{a}^{1}\left(i\right)=${$T_{max}$, *d*, *q*} and column (**b**) corresponds to the pattern vector $v_{a}^{1}\left(i\right)=${θ, *d*, *q*}. As can be seen, at the optimal position a=0.0168 m, the scattergrams show a correct separation between normal and abnormal thermograms. In other cases, the feature vectors are highly correlated.

**Figure 10:**
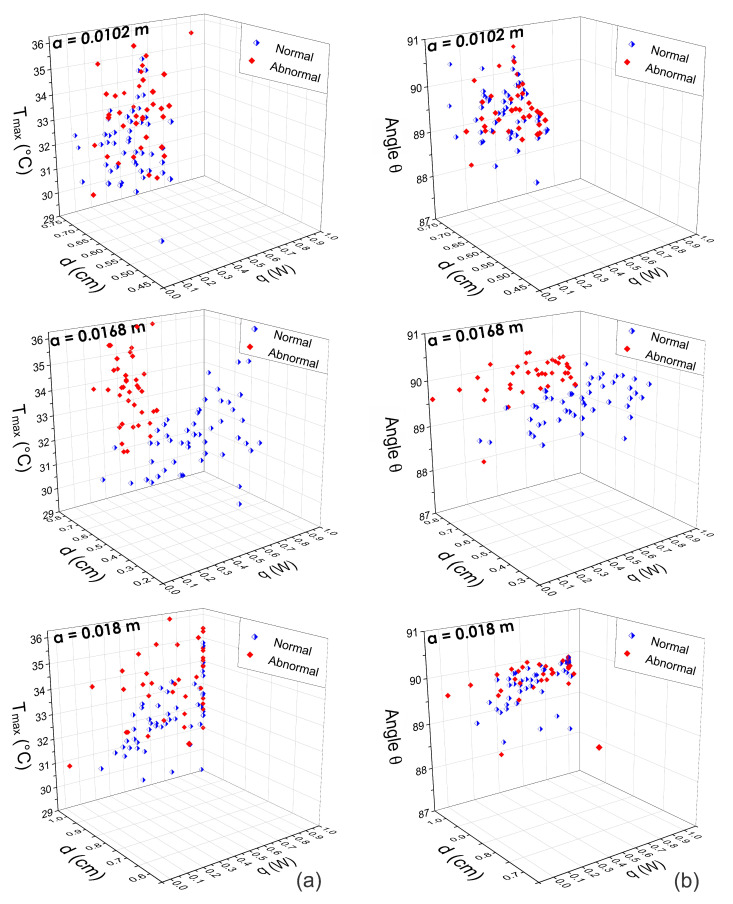
Three-dimensional scattergrams using the physiological parameters obtained by means of the D-I-R model at different positions a=0.0102 m, a=0.0168 m, and a=0.018 m. Column (**a**) corresponds to the pattern vector $v_{a}^{2}\left(i\right)=${$T_{max}$, *d*, *q*} and column (**b**) corresponds to the pattern vector $v_{a}^{2}\left(i\right)=${θ, *d*, *q*}. As can be observed, at the optimal position a=0.0168 m, the scattergram shows a correct separation between normal and abnormal thermograms. In other cases, the feature vectors are highly correlated.

**Figure 11:**
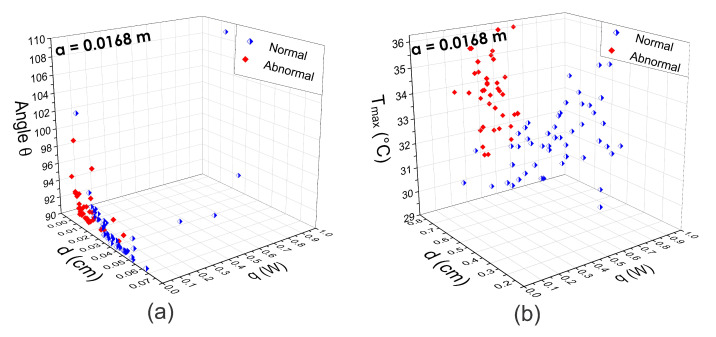
Three-dimensional scattergrams using the physiological parameters extracted from (**a**) the fitting method of Lorentz curve and (**b**) the D-I-R model. As can be seen, at the same optimal position a=0.0168 m, the scattergrams show a correct separation between normal and abnormal thermograms in both cases.

Thus, the internal heat source parameters *d*, *q*, and *R* are extracted from the obtained STD at different *a* positions. Figure 9, Figure 10 and Figure 11 show the three-dimensional feature spaces using the physiological parameters $T_{max}$, *d*, *q*, and θ extracted at different *a* positions. The heat source parameters were obtained (a) by fitting the temperature distribution with the Lorentz curve (n=1) and (b) the D-I-R model (n=2) from Equations (5)–(7). Scattergrams proof the correct separation between normal and abnormal thermograms at the optimal position a=0.0168 m that was determined using SVM. At different *a* positions, the physiological parameters are scattered from their respective cluster.

For the classification task, physiological pattern vectors composed of $v_{a}^{1,2}\left(i\right)=\;${$T_{max},d,q,R,\theta$} are used. Using SVM as a classifier, we obtain the CRC of Table 3. As can be observed, the higher classification percentages are obtained using the estimated pattern vector $v_{a=0.0168\;m}^{2}\;\left(i\right)$ based on the D-I-R model. In this case, the STD from the RoI indicates a good fitting with the estimated STD. The results are achieved using the proposed pattern vector extracted at an optimal a=0.0168 m position and through the SVM algorithm. In addition, we analyze the performance of the proposed methodology using the AUC of the Receiver Operating Characteristic (ROC) curve for the two methods employed.

**Figure 12:**
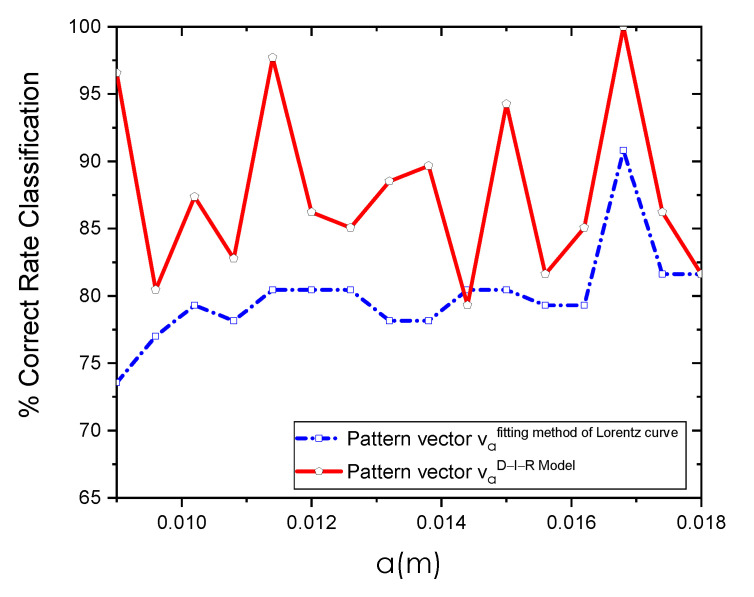
Classification results using the pattern vector $v_{a}^{n}\left(i\right)=${$T_{max},d,q,R,\theta$} obtained through the fitting method of Lorentz curve and D-I-R model at different *a* positions using SVM as a classifier.

Figure 12 shows the CRC percentages achieved at distinct *a* positions. As can be seen, the highest percentages using the pattern vectors $v_{a}^{1,2}\left(i\right)$ extracted with the fitting method of Lorentz curve and D-I-R model resulted in the same a=0.0168 m position.

Table 4 shows the accuracy, sensitivity, and specificity of the two methods used for the extraction of physiological parameters. These values indicate that the performance of the pattern vector $v_{a=0.0168\;m}^{1}\;\left(i\right)$ obtained employing the fitting method of Lorentz curve was lower than the results given by the pattern vector $v_{a=0.0168\;m}^{2}\;\left(i\right)$ calculated with the D-I-R model. These measures of diagnostic accuracy are obtained at optimal a=0.0168 m position using SVM.

**Figure 13:**
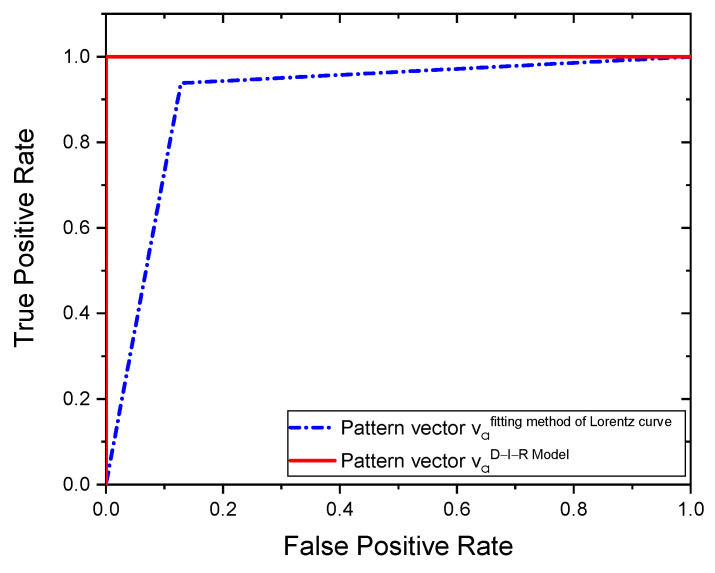
ROC curves.

The ROC curves from the two tested methods reached high performances, as shown in Figure 13. On the other hand, the area under ROC confirms that the proposed pattern vector $v{{}_{a}}^{2}\left(i\right)$ has better performance since it has an area of 1, followed by the proposed vector $v{{}_{a}}^{1}\left(i\right)$ with an area of 0.9046. In this study, for the pattern vectors $v_{a=0.0168\;m}^{1,2}\;\left(i\right)$ obtained with the fitting method of Lorentz curve and the D-I-R model, we achieve higher performance CRC rates of 90.80% and 100%, respectively, and an area under the ROC curve of 0.9046 and 1 for the two methods employed.

## 4. Discussion

An analytical-based solution for the thermal inverse problem was used, considering a point heat source embedded in tissue. From this solution and using the D-I-R model, the physiological parameters, *q*, *d*, and *R* of an internal heat source are estimated. During thermal analysis of breast thermograms from the DMR-IR, we observe that several factors can affect the accuracy of the extracted parameters, such as imaging acquisition conditions, localization of the hottest spots, and the radial extension *a* of the RoI. Despite these drawbacks, our experimental results show that the proposed method can classify breast thermograms without intensity preprocessing or normalization of the raw thermal data. The three-dimensional feature space in Figure 9, Figure 10 and Figure 11 supports the discrimination power of the proposed pattern vectors $v_{a=0.0168\;m}^{1,2}\;\left(i\right)=${$T_{max},d,q,R,\theta$}, allowing the correct separation of both classes according to the optimal *a* position. Each normal and abnormal class forms a cluster, and they are well separated. The proposed pattern vectors $v_{a}^{1,2}\;\left(i\right)=$ {$T_{max},d,q,R,\theta$} are obtained using the fitting method of Lorentz curve and the D-I-R model. We can see that at position a=0.0102 m and a=0.018 m, descriptors values are widely correlated. Because of that, we determine the optimal position as a=0.0168 m through the SVM algorithm. The physiological parameters extracted at this position are suitable for breast thermogram classification, despite some descriptors in the 3D scattergrams that have been sparse from their respective class due to the human body complex and the inherent nature of the acquisition data. Figure 11 shows that the highest classification percentages are obtained when the physiological pattern vectors are extracted at the position a=0.0168 m. We find that the optimal position is a=0.0168 m for both the fitting method of Lorentz curve and the D-I-R model, with a CRC of 90.80% and 100%, respectively, by using the proposed pattern vector.

On the other hand, the DMR-IR database has been used to evaluate the efficiency of the most common extracted features such as texture [19,28,29,30,31], shape [32], and morphology [33] descriptors to classify thermal patterns. However, the discriminative power of the physiological descriptors has not been evaluated on the above-mentioned database. Table 5 summarizes research that aims to classify breast tumors into two categories: benign and malignant. As can be seen, the classification percentages below 97.18% are reached using the DMR-IR database with a maximum of 80 thermograms. In this work, we proposed highly discriminative physiological pattern vectors $v_{a=0.0168\;m}^{1,2}\;\left(i\right)$ for breast thermogram categorization. Furthermore, our proposed method uses a minimal number of descriptors to obtain a CRC of 100% when 87 thermograms are used.

## 5. Conclusions

Based on an inverse solution of the bio-heat equation and using the surface temperature distribution of the RoI, the physiological parameters of an input heat source are estimated using the fitting method of Lorentz curve and the D-I-R model. In this research, we analyze i=1,…,87 breast thermograms from DMR-IR with clinically confirmed cases as sick or healthy. Highly discriminative proposed pattern vectors were extracted, and they are composed of physiological parameters $v_{a=0.0168\;m}^{1,2}\;\left(i\right)=$ {$T_{max},d,q,R,\theta$} for breast thermogram classification. The pattern vector employing the D-I-R model is able to classify when the parameters are extracted at an optimal *a* position. As can be seen in Table 3, we obtain a CRC of 100% using SVM as a classifier. According to the achieved results, we found that the optimal position a=0.0168 m is suitable for the thermal analysis using both the fitting method of Lorentz curve and the D-I-R model. Furthermore, the proposed technique utilizes a reduced number of physiological parameters $|v_{a}^{n}|=5$, and it does not apply any image normalization or contrast improvement. Our proposed method allows delimitation of the RoI using SVM for analysis and raw thermal pattern classification. Nevertheless, the experimental radial distance *a* of the RoI is less than or equal to 1.8 cm due to maximum the temperature location close to the boundary image. In a future work, we will use lateral view breast thermograms to overcome this limitation and analyze the whole breast region, including the armpit and lymph nodes. Thus, this method will be greatly valuable for determining the size and description of the RoI when it shows a pathological change not only in infrared imaging of the breast but also in the legs thermograms, abdomen, arms, and head.

## Figures and Tables

**Table 1:** Thermal and physical parameters of tumor.

*T* (°C)	Surface temperature distribution.
ρ (Kg/m^3^)	Biological tissue’s density.
*c* (J/Kg · °C)	Thermal capacity of biological tissue.
*k* (W/m/°C)	Heat conduction coefficient.
$w_{b}$ (Kg/m^3^ · s)	Blood perfusion rate.
$\rho_{b}$ (Kg/m^3^)	Blood density.
$c_{b}$ (J/Kg · °C)	Blood thermal capacity.
$T_{a}$ (°C)	Arterial blood temperature.
$Q_{m}$ (W/m^3^)	Metabolic heat rate.
*q* (W)	Heat source intensity.
*d* (cm)	Heat source depth.
*R* (m)	Radius of spherical heat source.
*a* (m)	Distance from point $O^{\prime }$ to an arbitrary point on the body surface.
*r* (m)	Distance from point *O* to an arbitrary point on the body surface.
*O*	Point heat source position.
$O^{\prime }$	The hottest spot of the RoI.
$T_{max}$ (°C)	Maximum temperature.
$h_{0}$ (W/m^2^ · °C)	Heat exchange coefficient.
$T_{e}$ (°C)	Ambient temperature.
θ, ψ (degrees)	Spherical coordinates.
$\mu_{prom}$	Mean temperature.
$t_{min}$	Minimum temperature.

**Table 2:** Technical specifications of the FLIR SC−620 sensor [4,17].

Image resolution M×N	640×480 pixels
Pixel size	45 μm
Sensor size	2.88 cm × 2.16 cm
Standard temperature range	−40 °C to +500 °C
Sensitivity	<0.04 °C

**Table 3:** Results of CRC and Area Under Curve (AUC) using the proposed pattern vector composed of physiological parameters.

Method	Breast Thermograms	R-Squared	CRC	AUC	Optimal Position of the RoI
D-I-R Model	87	0.9999	100%	1	a=0.0168 m
Fitting method of Lorentz curve	87	0.87	90.80%	0.9046	a=0.0168 m

**Table 4:** Measures of accuracy.

Method	Accuracy	Sensitivity	Specificity
D-I-R model	100%	100%	100%
Fitting method of Lorentz curve	90.8%	87%	97%

**Table 5:** Summary of research works that make use of the DMR-IR.

Authors	Segmentation	Features Extracted	CRC	Thermograms Number
Sathish et al. [19]	The breast is segmented.	Histogram and Gray Level Cooccurrence Matrix (GLCM) -based texture features.	90%	80
R. Devi et al. [28]	The left and right breast are separated.	GLCM features and first-order histogram.	95%	60
V. Mishra [30]	The breast is segmented.	Gray Level Run Length Matrix (GLRLM) and GLCM.	95.45%	56
U. R. Gogoi [31]	The breast is segmented.	First-order statistical features.	—	60
S. S. Suganthi et al. [32]	The breast is segmented.	Anisotropy and orientation measures.	—	20
R. Resmini et al. [34]	The breast is segmented with different approaches (with and without armpits) to compose four experiments.	GLCM, Local Ternary Pattern, Daubechies Wavelet, Higuchi, Petrosian Fractal, Dimensions, and Hurst Coefficient.	97.18%	80
Proposed approach	The breast is segmented with a well-defined RoI using SVM.	Physiological pattern vectors $v_{a=0.0168m}^{1,2}\;\left(i\right)$ = { $T_{max}$, *q*, *d*, *R*, θ}.	100%	87

## Data Availability

All data used in this research were obtained from breast thermographic dataset DMR-IR and are completely available at http://visual.ic.uff.br/dmi (accessed on 1 March 2021).

## References

[1] Fernández C.I., Bouzas J., Arnáiz J., Gómez P., Piñonosa C., García M., Sillero M. (2015). Classification of factors influencing the use of infrared thermography in humans: A review. Infrared Phys. Technol..

[2] Lawson R., Chughtai M.S. (1963). Breast Cancer and Body Temperature. Can. Med. Assoc. J..

[3] Silva L.F., Saade D.C., Sequeiros G.O., Silva A.C., Paiva A.C., Bravo R.S., Conci A. Database for Mastology Research with Infrared Image (DMR-IR). http://visual.ic.uff.br/dmi.

[4] Silva L.F., Saade D.C., Sequeiros G.O., Silva A.C., Paiva A.C., Bravo R.S., Conci A. (2014). A new database for breast research with infrared image. J. Med. Imaging Health Inform..

[5] Pennes H.H. (1998). Analysis of tissue and arterial blood temperatures in the resting human forearm. J. Appl. Physiol..

[6] Hossain S., Mohammadi F.A., Nejad E.T. Neural network approach for the determination of heat source parameters from surface temperature image. Proceedings of the 2011 24th Canadian Conference on Electrical and Computer Engineering (CCECE).

[7] Chunfang G., Kaiyang L., Shaoping Z. A Novel Approach of Analyzing the Relation between the Inner Heat Source and the Surface Temperature Distribution in Thermal Texture Maps. Proceedings of the Annual International Conference of the IEEE Engineering in Medicine and Biology Society.

[8] Shi G., Han F., Wang L., Liang C., Li K. (2015). Q-r curve of thermal tomography and its clinical application on breast tumor diagnosis. Biomed. Opt. Express.

[9] Shi G., Wang L., Han F., Liang C., Li K. (2015). Diagnosis of breast tumor using thermal tomography *q*-*r* curve. J. Biomed. Opt..

[10] Rastgar-Jazi M., Mohammadi F. (2017). Parameters sensitivity assessment and heat source localization using infrared imaging techniques. BioMed. Eng. Online.

[11] Han F., Shi G., Liang C., Wang L., Li k. (2015). A Simple and Efficient Method for Breast Cancer Diagnosis Based on Infrared Thermal Imaging. Cell Biochem. Biophys..

[12] Minhua Z., Qian C. Study of the Surface Temperature Distribution of the Tissue Affected by the Point Heat Source. Proceedings of the 2007 1st International Conference on Bioinformatics and Biomedical Engineering.

[13] Zermeño L.O., Orozco E., Toxqui C., Padilla A., Mejias Y.N. (2017). Caracterización de imágenes en la región espectral del infrarrojo para la detección de lesiones en mama. Rev. Inst. TecnolóGico Cd. JuáRez Acad. J..

[14] Cristianini N., Taylor J.S. (2000). An Introduction to Support Vector Machines and Other Kernel-Based Learning Methods.

[15] Farooq M.A., Corcoran P. Infrared Imaging for Human Thermography and Breast Tumor Classification using Thermal Images. Proceedings of the 2020 31st Irish Signals and Systems Conference (ISSC).

[16] Silva T.A.E.D., Silva L.F.D., Muchaluat-Saade D.C., Conci A. (2020). A computational method to assist the diagnosis of breast disease using dynamic thermography. Sensors.

[17] FLIR Systems FLIR SC620 Infrared Camera Datasheet FLIR Systems. http://w1.sayato.com/7040/file/FLIR%20SC620.pdf.

[18] Castro R., Toxqui C., Padilla A., Solís J. F., Orozco E. E. (2019). Zernike moment invariants for hand vein pattern description from raw biometric data. J. Electron. Imaging.

[19] Sathish D., Kamath S., Prasad K., Kadavigere R., Roshan J. (2017). Asymmetry analysis of breast thermograms using automated segmentation and texture features. Signal Image Video Process..

[20] Garduño R.M.A., Vega M.S.G., Morales H.L.A., Osornio R.R.A. (2017). Supportive Noninvasive Tool for the Diagnosis of Breast Cancer Using a Thermographic Camera as Sensor. Sensors.

[21] Alvarado L.B., Toxqui C., Hernández J.A., Padilla A. Breast thermography: A non-invasive technique for the detection of lesions. Proceedings of the Applications of Digital Image Processing XLI.

[22] STD Plugin Computer Vision Laboratory. https://sites.google.com/view/lvc-upt/inicio.

[23] Cortes C., Vapnik V. (1995). Support-vector networks. Mach. Learn..

[24] Karatzoglou A., Meyer D., Hornik K. (2006). Support vector machines in R. J. Stat. Softw..

[25] Lin W., Yuan X. (2004). Classification of in vivo autofluorescence spectra using support vector machines. J. Biomed. Opt..

[26] Castro J., Toxqui C., Manriquez F., Orozco E., Padilla A., Sánchez J.J. Detecting Jaundice by using digital image processing. Proceedings of the Three-Dimensional and Multidimensional Microscopy: Image Acquisition and Processing XXI.

[27] Steel R.G., Torrie J.H. (1960). Principles and Procedures of Statistics with Special Reference to the Biological Sciences.

[28] Devi R.R., Anandhamala G.S. (2019). Analysis of breast thermograms using asymmetry in infra-mammary curves. J. Med. Syst..

[29] Acharya U.R., Ng E.Y.K., Tan J.H., Sree S.V., Saade D.M., Conci A. (2012). Thermography Based Breast Cancer Detection Using Texture Features and Support Vector Machine. J. Med. Syst..

[30] Vartika M., Rath S.K. (2020). Detection of breast cancer tumours based on feature reduction and classification of thermograms. Quant. Infrared Thermogr. J..

[31] Gogoi U.R., Majumdar G., Bhowmikl M.K., Ghoshl A.K., Bhattacharjee D. Breast abnormality detection through statistical feature analysis using infrared thermograms. Proceedings of the 2015 International Symposium on Advanced Computing and Communication (ISACC).

[32] Suganthi S.S., Ramakrishnan S. (2014). Analysis of breast thermograms using gabor wavelet anisotropy index. J. Med. Syst..

[33] Alvarado L.B., Delgadillo M., Toxqui C., Padilla A., Castro R., Arreola M.M. (2019). Fractal analysis for classification of breast lesions. Proceedings of the Current Developments in Lens Design and Optical Engineering XX.

[34] Resmini R., Silva L., Araujo A.S., Medeiros P., Saade D.M., Conci A. (2021). Combining Genetic Algorithms and SVM for Breast Cancer Diagnosis Using Infrared Thermography. Sensors.

